# Disorders of Sex Development in a Large Ukrainian Cohort: Clinical Diversity and Genetic Findings

**DOI:** 10.3389/fendo.2022.810782

**Published:** 2022-03-21

**Authors:** Evgenia Globa, Natalia Zelinska, Yulia Shcherbak, Joelle Bignon-Topalovic, Anu Bashamboo, Ken MсElreavey

**Affiliations:** ^1^ Ukrainian Scientific and Practical Center of Endocrine Surgery, Transplantation of Endocrine Organs and Tissues of the Ministry of Health of Ukraine, Kyiv, Ukraine; ^2^ National Children’s Specialized Hospital OHMATDYT of the Ministry of Health of Ukraine, Kyiv, Ukraine; ^3^ Human Developmental Genetics, Institute Pasteur, Paris, France

**Keywords:** 46,XY and 46,XX disorders of sex development, genes, karyotype, phenotype, whole exome sequencing (WES)

## Abstract

**Background:**

The clinical profile and genetics of individuals with Disorders/Differences of Sex Development (DSD) has not been reported in Ukraine.

**Materials and Methods:**

We established the Ukrainian DSD Register and identified 682 DSD patients. This cohort includes, 357 patients (52.3% [303 patients with Turner syndrome)] with sex chromosome DSD, 119 (17.5%) with 46,XY DSD and 206 (30.2%) with 46,XX DSD. Patients with sex chromosome DSD and congenital adrenal hyperplasia (CAH, n=185) were excluded from further studies. Fluorescence *in situ* hybridization (FISH) was performed for eight 46,XX boys. 79 patients underwent Whole Exome Sequencing (WES).

**Results:**

The majority of patients with 46,XY and 46,XX DSD (n=140), were raised as female (56.3% and 61.9% respectively). WES (n=79) identified pathogenic (P) or likely pathogenic (LP) variants in 43% of the cohort. P/LP variants were identified in the androgen receptor (*AR*) and *NR5A1* genes (20.2%). Variants in other DSD genes including *AMHR2, HSD17B3, MYRF, ANOS1, FGFR11, WT1, DHX37, SRD5A1, GATA4, TBCE, CACNA1A* and *GLI2* were identified in 22.8% of cases. 83.3% of all P/LP variants are novel. 35.3% of patients with a genetic diagnosis had an atypical clinical presentation. A known pathogenic variant in *WDR11*, which was reported to cause congenital hypogonadotropic hypogonadism (CHH), was identified in individuals with primary hypogonadism.

**Conclusions:**

WES is a powerful tool to identify novel causal variants in patients with DSD, including a significant minority that have an atypical clinical presentation. Our data suggest that heterozygous variants in the *WDR11* gene are unlikely to cause of CHH.

## Highlights

•*What is already known on this topic?* DSD is a group of rare conditions that are defined by a discordance of the chromosomal, gonadal or phenotypic features. The genetic cause is known in a minority of patients and obtaining a genetic etiology is challenging due to a variable clinical presentation. A small number of studies have applied WES to large cohorts of DSD.

•*What does this study add?* This study describes the main clinical features and genetic findings in a large cohort of DSD patients from Ukraine. The most common genetic causes of DSD were variants in the *AR* and *NR5A1* genes. A significant number (35.3%) of patients with a genetic diagnosis had an atypical clinical presentation. A variant in *WDR11*, previously reported to cause CHH, was identified in individuals with primary hypogonadism suggesting that heterozygous variants in this gene may not always cause CHH.

## Introduction

Disorders of Sex Development (DSD) is a group of rare conditions that are defined by a discordance of the chromosomal, gonadal or phenotypic features of the internal and/or external genitalia (1). The development of the gonads is a complex process governed by a combination of genetic networks and hormonal signaling. Given the complexity of gonad formation and differentiation, comprehensive genetic testing is recognized as a key element in the investigation of patients with DSD (2). On the basis of the underlying etiology, DSD can be further divided into several subclasses, such as primary disorders of gonadal development, hormone secretion or hormone action and syndromic conditions (1).

The most common cause of 46,XY DSD is the disruption of sex hormone synthesis or anomalies of their receptors, such as variants in the androgen receptor (AR). Approximately 30-45% of patients with partial androgen insensitivity syndrome (PAIS) carry pathogenic variants in the AR (3, 4). In contrast, more than 80% of 46,XY DSD raised as females, have pathogenic variants in the *AR* gene (4). Anomalies of testis-determination result in gonadal dysgenesis and are usually caused by the pathogenic variants in the *SRY*, *NR5A1* and *DHX37* genes (15%, 10-13% and 10-12% respectively) (4–6). Pathogenic variants in the *MAP3K1*, *MAMLD1, HSD17B3, SRD5A2* and *DAX1* genes are relatively infrequent (5-10%) (7, 8). The most common presentation of 46,XX DSD is congenital adrenal hyperplasia (CAH) that is caused by pathogenic variants in the *CYP21A2* gene (9). Translocation of the testis-determining gene *SRY* from the Y chromosome to the X chromosome is responsible for up to 90% of cases of 46,XX testicular or ovotesticular DSD (10).

Defining the genetic causes of DSD using a gene by gene approach can identify the molecular etiology in up to 64% of cases (11, 12). Targeted next generation sequencing (tNGS) gene panels can result in a diagnostic yield of 43% for 46,XY DSD (13). Excluding patients with CAH, pathogenic variants in the *AR, NR5A1, SRD5A2, ZFPM2, HSD17B3* and *DHH* genes are the most frequent causes of 46,XY DSD (13). However, since the underlying genetic etiology of DSD can vary depending on geography and ancestry, the diagnostic yield may differ from one region to another. Whole Exome Sequencing (WES) can overcome these problems by theoretically sequencing all of the genes in the human genome. It also allows data to be reanalyzed as new genetic causes are identified. Currently, more than 60 genes that cause DSD are known (5, 14). A genetic diagnosis allows further knowledge-based clinical management as well as genetic counseling on variant transmission, fertility and the risk of malignancy. Despite the power of WES, the interpretation and reporting of genetic variants is challenging and secondary findings that are not related to the primary condition may arise. Since a small number of studies have used WES on large DSD cohorts, the aim of this study was to determine the main clinical features and the genetic findings of a large cohort of DSD patients from Ukraine.

## Materials and Methods

### Patients

The Ukrainian DSD Registry was created in 2014 to include children diagnosed with DSD, identified by regional Ukrainian pediatric endocrinologists, gynecologists, urologists and contains 682 patients. Chromosomal DSD was found in 357 patients (52.3%, of which 303 patients had Turner syndrome (84.8%)). In the 46,XX DSD group (n=206, 30.2%) CAH was diagnosed in 185 patients (89.8%). We also identified 140 cases (20.5%) with 46,XY (n=119) or 46,XX DSD (n=21). Of these DNA samples were obtained from 79 patients (56.4%) from 75 unrelated families for the further genetic testing using WES.

In this cohort study the diagnosis of DSD was done based on clinical evaluation, laboratory and imaging examination in according to the Consensus Statement on Management of Intersex Disorders (1). Before WES all patients underwent routine clinical examination, hormonal tests and instrumental diagnostics (including assessment of bone age, ultrasound (US) and/or MRI if necessary). For patients who undergone gonadectomy, additional data included - age at gonadectomy, indication for this procedure and histology result. All patients had a karyotype determined by standard methods, and for eight 46,XX boys, molecular cytogenetic studies (FISH) was done to determine a Y-X translocation of the *SRY* locus.

The main inclusion criteria included the following - ambiguous external genitalia (female genitalia with an enlarged clitoris, posterior labial fusion, or an inguinal/labial mass and/or inguinal hernia or male genitalia with bilateral undescended testes, micropenis, isolated perineal hypospadias, or mild hypospadias with undescended testis), delayed or incomplete puberty, virilization with typical female external genitalia, primary amenorrhea, breast development in a typical male, a discordance between the genital appearance and the karyotype and family history of DSD (1). Patients with specific chromosomal DSD anomalies (e.g., Turner syndrome, Klinefelter syndrome etc.) and those with CAH were excluded from further study.

The clinical presentation of 46,XY DSD (n=71) patients who underwent WES included (i) DSD of undefined origin (n=22, including 16 patients with severe ambiguous genitalia (AG) at birth) with a broad spectrum of phenotypes for which the underlying cause was unknown; (ii) a suspected disorder in androgen synthesis or action (DASA) [n=18, including 2 patients with Persistent Müllerian Duct Syndrome (PMDS)]; (iii) confirmed or probable gonadal dysgenesis (GD) (n=14); (iv) testicular regression syndrome (TRS) (n=12); (v) Kallmann syndrome (n=4) and (vi) a patient with 46,XY ovotesticular DSD (n=1). The 46,XX patients (n=8) who underwent WES, consisted of five girls with a primary hypogonadism (PH) without virilization, whilst two had signs of androgen excess (Prader 3-4) and one phenotypic male who presented with testicular DSD.

### Genetic Testing

Exome sequencing of genomic DNA was performed for 79 patients. Enrichment for WES was generated with Agilent SureSelect Human All Exon V4, followed by paired-end sequencing on the Illumina HiSeq2000 platform with TruSeq v3 chemistry. Data analysis was performed from the sequencing platform using manufacturer’s proprietary software. All reads were aligned against the human reference genome (NCBI, GRCh37/hg19 or GRCh38/hg38) *via* Burrows-Wheeler aligner. Single-nucleotide variants and small insertions and deletions (InDel) were selected with GATK version 1.6. Picard version 1.62 (http://broadinstitute.github.io/picard/) and SAMtools version 0.1.18 were used to mark duplicate reads and to process the BAM files manipulations, respectively. For each case, single-nucleotide polymorphism (SNP) and indel variants were annotated to dbSNP 138 identifiers using the Genome Analysis Toolkit (GATK) Unified Genotyper. The SNP Effect Predictor bioinformatics tools on the Ensembl website (http://www.ensembl.org/homosapiens/userdata/uploadvariations), gnomAD (https://gnomad.broadinstitute.org/) and ClinVar (https://www.ncbi.nlm.nih.gov/clinvar/) were used to annotated the novel variants, followed by manual screening of all variants by using the Human Gene Mutation Database Professional Biobase (http://www.biobaseinternational.com/product/hgmd/). Potentially pathogenic variants were confirmed by Sanger sequencing.

## Results

**Figure 1:**
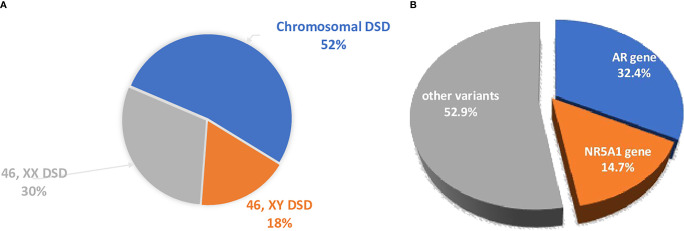
**(A)** The distribution of the different forms of DSD in the Ukrainian DSD registry. 46,XX DSD cohort included 89.8% CAH patients. **(B)** The distribution of the main genetic causes of DSD were analyzed by WES. The dominant causes are due to variants in the *AR* and *NR5A1*.

The Ukrainian DSD Registry has 682 patients, consisting of 357 (52.3%) individuals with chromosomal DSD, 119 (17.5%) individuals with 46,XY DSD and 206 patients with 46,XX DSD (30.2%), (**Figure 1A**). Molecular genetic studies (WES) were performed in a selected group of patients (n=79) from 75 unrelated families with 46,XY and 46,XX DSD.

All patients included in the study, underwent follow-up examinations and have been fully analyzed. The majority of affected individuals included in the WES study, were raised as female (56.3% 46,XY DSD and 61.9% 46,XX DSD). P/LP variants were identified in 34/79 patients (43%), however two patients had P variants that could either not explain all of the phenotype, or the phenotypic expression was atypical for the gene (**Table 1**). Of the thirty-four patients carrying P/LP variants, twenty-eight patients (35.4%) carried variants that have not been reported in association with the pathology, nor have they been reported in any public variant database [variants of unknown significance (VUS)].

**Table 1:** Clinical details of the individuals with Pathogenic or Likely Pathogenic variants causing DSD identified by WES.

	Variant	Sex of rearing	Age at presentation	External genitalia	Position of gonads	Preliminary clinical diagnosis	Somatic anomalies	Gonadectomy (age, histology)
1	*AR* p.I836S	f	2 yr	Female	Inguinal canals	46XY, CAIS	Pigmented nevi	14 yr., testicular tissue with fibrosis and foci of accumulation of Leydig cells in the interstitium. In the testicular tissue and around it an adenomatoid tumor (benign non-papillary mesothelioma) and a microtumor from Leydig cells, represented by compact clusters. Hypoplastic epididymis with cystic dilated ducts of the epididymis hydatid.
2	*AR* p.N706S	f	birth	Bilateral inguinal hernias, vaginal aplasia	Inguinal canals	46XY, CAIS	no	5 yr., testicular fibrosis
3	*AR* p.N706S	f	6 yr	Female	Inguinal canals	46XY, CAIS	no	14 yr., testicular sertolioma
4	*AR* p.H886L	f	birth	Female, clitoromegaly	Inguinal canals	46XY, PAIS	Growth delay	No
5	*AR* p.Q799E	m	birth	Left inguinal hernia, perineal hypospadias, micropenia	Inguinal canals	46XY, PAIS	no	No
6	*AR* c.2607+2T>G	f	13 yr	Inguinal hernia at 10 M, relapse at 14 M. Vaginal aplasia	both gonads – near the uterus	46XY, CAIS	no	15 yr., areas of the testicle with pronounced interstitial fibrosis and the presence of immature fetal structures. Dysgenetic gonad. Immunohistochemical indicated glomus angioma
7	*AR* p.I843T (sibling of pt 8)	m	4 M	Micropenia, testis hypoplasia	Scrotum	46XY, PAIS	Gynaecomastia at 14 yr	No
8	*AR* p.I843T (sibling of pt 7)	f	20 M	Clitoromegaly and urogenital sinus	Scroto-labial folds	46XY, PAIS	no	4 yr., clitorectomy, reconstruction of the urogenital sinus. 13 yr., testicular tissue with pronounced interstitial edema. Rudimentary epididymis
9	*AR* p.Leu839Ile	m	birth	Ambiguous genitalia, perineal hypospadias, bilateral cryptorchidism	Inguinal canals	46XY, PAIS	Gynaecomastia at 13 yr	No
10	*AR* p.Arg841Cys	m	birth	Perineal hypospadias, bilateral cryptorchidism	abdominal cavity, left sided TRS	46XY, PAIS	Gynaecomastia at 10 yr	No
11	*AR* p.Arg775His	f	15 yr	Female, vaginal hypoplasia	Inguinal canals	46XY, CAIS	no	16 yr., dysgenetic gonad of the type of immature testicle
12	*NR5A1*, c.244+1G>T (sibling of pt 13)	m	birth	Bilateral cryptorchidism, perineal hypospadias and micropenia, hypoplasia of the corpora cavernosa, hypoplasia of the scrotum	Left gonad in the inguinal canal, right – in the abdominal cavity	46XY, U	no	No
13	*NR5A1*, c.244+1G>T (sibling of pt 12)	m	birth	Bilateral cryptorchidism, perineal hypospadias and micropenia, hypoplasia of the corpora cavernosa, hypoplasia of the scrotum	Right gonad in the inguinal canal, left – in the abdominal cavity	46XY, U	no	No
14	*NR5A1*, p.C73Y	f	birth	Ambiguous genitalia, urogenital sinus, clitoromegaly	Migratory gonads: from abdominal position to scrotolabial folds	46XY, U, AG	Chronic pyelonephritis, kidney pyelectasis. Hemorrhoids at 4 yr	10 yr., fragments of tissue of the testicle and epididymis with edema.
15	*NR5A1*, p.G35D	f	birth	Clitoromegaly and urogenital sinus. Right-sided inguinal hernia at 3 yr	Migratory gonads: from abdominal position to the inguinal canals	46XY, U	Arnold Chiari anomaly, Mild adrenal insufficiency	10 yr., dysgenetic gonads
16	*NR5A1*,p.C73W	f	birth	Clitoromegaly at 3 M. Urogenital sinus	Inguinal canals	46XY, U	Intellectual disability	No
17	*AMHR2*, p.R463H/R471H (sibling of pt 18)	m	birth	Glanular hypospadias and bilateral cryptorchidism	Abdominal cavity	46XY, PMDS	no	10 yr., biopsy: loose connective tissue, fetal cord-like structures with immature tubule
18	*AMHR2*, p.R463H/R471H (sibling of pt 17)	m	birth	Bilateral inguinal cryptorchidism and coronal hypospadias	Inguinal canals	46XY, PMDS	no	No
19	*HSD17B3*, p.E215D/ c.277+4A>T	f	7 M	Bilateral inguinal hernias, hernia repair at 6M, relapse at 2 yr	Inguinal canals	46XY, DASA	no	12 yr., dysgenetic gonads with morphological signs of immaturity (fetal tubular differentiation present). Elements of the adenomatoid tumor in the left gonad.
20	*HSD17B3*, p.M47V/p.V243fs	f	birth	Ambiguous genitalia (scroto-labial folds, perineal hypospadias, urogenital sinus, clitoromegaly)	Migratory gonads: 1 yr in scroto-labial folds; 2 yr in the abdominal cavity; 3 yr in scroto-labial folds; 6 yr - Inguinal canals	46XY, U, AG	no	6 yr – clitorectomy, gonadectomy. The gonads have a prepubertal testicular structure
21	*MYRF*, p.N105D	m	birth	Ambiguous genitalia, perineal hypospadia, bilateral cryptorchidism	Left testis - inguinal canal; right testis – abdominal cavity.	46XY, U, AG	no	Testicular biopsy confirmed the presence of testicular tissue with severe stromal sclerosis and tubular atrophy
22	*MYRF*, c.2572+1G>A	f	14 yr	Clitoromegaly, vaginal hypoplasia	One migratory gonad from abdominal cavity to inguinal canal	46XY, GD	High-grade hypermetropia	26 yr., gonadectomy: lack of germ cells, presence of Sertoli cells and Leydig cell hyperplasia 29 yr., vaginoplasty and clitorectomy
23	*ANOS1*, p.Gln586	m	17 yr	Hypogenitalism	Scrotum	46XY, HH	Hyposmia, renal cysts	No
24	*ANOS1*, p.Gln57fs* (cousin of pt 25)	m	6 yr	Bilateral inguinal cryptorchidism	Inguinal canals	46XY, HH	Hyposmia, hypermetropic astigmatism of both eyes.	No
25	*ANOS1*, p.Gln57fs*	m	1 yr	Right-sided inguinal cryptorchidism	Inguinal canals	46XY, HH	Hyposmia	No
26	*FGFR1* c.179_208del; p.Asp60_Asp69del	m	birth	Micropenia since birth. Persistent bilateral inguinal cryptorchidism at 4 yr, micro-orchidism	Inguinal canals	46XY, HH	Anosmia, pulmonary hypoplasia, epilepsy, bronchiectasis, primary hypothyroididsm, hypoparathyroidism	No
27	*WT1*, p.S255L	f	12 yr	Typical female genitalia at birth	Migratory gonads: inguinal canals; under the skin of the pubis	46XY, GD	no	12 yr., fetal gonadal tissue differentiation by tubular sex cord stromal cells with signs of Leydig cells and Sertoli cells, immature gonad with impaired maturation of the elements of stroma of the genital tract
28	*DHX37*, p.R674Q	f	3 yr	Inguinal hernia at 3 yr, urogenital sinus	Abdominal cavity	46XY, GD	Bilateral vesicoureteral reflux, ureterohydronephrosis	4 yr., fibrosis, structures similar to ducts, fragments of the uterine tube with sclerosis
29	*SRD5A2*, p.Y91H/p.G13R	f	14 yr	Clitoromegaly, urogenital sinus	Migratory gonads: from inguinal canals to scroto-labial folds	46XY, DASA	no	14 yr., gonadectomy, reconstruction of the urogenital sinus and clitoris. Testicular tissue with the centers of clusters of Leydig cells and tissue of the epididymis, the pieces of the corpora cavernosa
30	*GATA4*, p.G12R	f	3 yr	Typical female genitalia	Abdominal cavity	46XY, GD	Dwarfism	4 yr., tissue of hypoplastic gonads and fallopian tubes. No signs of cellular atypia.
31	*TBCE*, c.100+1G>A	m	birth	Inguinal bilateral cryptorchidism	Inguinal canals	46XY, TRS	Cognitive impairment, dysmorphic features and recurrent infections, dwarfism	4 yr., atrophic changes in the left testicle 15 yr., atrophic and sclerotic changes in the right testicle, no signs of spermatogenesis
32	*CYP19A1*, p.R457X/ p.L353fs	f	birth	Prader 4, ambiguous genitalia, urogenital sinus	Abdominal cavity	46XX, U, AG	No	No
33	*CACNA1A*, p.I219V	m	birth	Bilateral cryptorchidism, scrotum hypospadias, micropenia	Abdominal cavity	46XY, ovotesticular DSD	Double left kidney, epilepsy	6 and 7 yr., germinogenic tumor
34	*GLI2*, p.E1577K	f	12 yr	Vaginal hypoplasia	Abdominal cavity	46XY, GD	Coarctation of the aorta, cleft palate, and facial dysmorphia, dwarfism	13 yr., fragments of fibrous tissue with structures similar to ducts with the presence of calcification. Fragments of the fallopian tube tissue. Structures similar to epididymis tissue.15 yr., bicornuate uterus with non-communicating rudimentary horn

m, male; f, female; yr, year; M, month; 46XY,U, undefined, 46XY,U, AG, undefined with AG.

### 46,ХY DSD Cohort

The main genetic causes of DSD in the XY cohort that underwent WES are pathogenic variants in the *AR* and *NR5A1* genes (16/79, 20.2%; **Tables 1**, **2** and **Figure 1B**).

**Table 2:** Genetic causes of DSD in patients from the Ukraine DSD Register.

Gene	Patient	Variant	Zygosity	MAF (population)	ClinVar (Variation ID)	Pathogenicity (ACMG)
*AR*	1	p.I836S	Hemi	Novel	NA	Pathogenic
*AR*	2	p.N706S	Hemi	Novel	NA	Pathogenic
*AR*	3	p.N706S	Hemi	Novel	NA	Pathogenic
*AR*	4	p.H886L	Hemi	Novel	NA	Pathogenic
*AR*	5	p.Q799E	Hemi	0.002741 (European)	Likely pathogenic (9846)	Likely Pathogenic
*AR*	6	c.2607+2T>G	Hemi	Novel	NA	Pathogenic
*AR*	7, 8 (siblings)	p.I843T	Hemi	Novel	NA	Pathogenic
*AR*	9	p.Leu839Ile	Hemi	Novel	NA	Likely Pathogenic
*AR*	10	p.Arg841Cys	Hemi	Novel	NA	Pathogenic
*AR*	11	p.Arg775His	Hemi	Novel	NA	Pathogenic
*NR5A1*	12, 13 (siblings)	c.244+1G>T	Het	Novel	NA	Pathogenic
*NR5A1*	14	p.C73Y	Het	Novel	NA	Pathogenic
*NR5A1*	15	G35D	Het	Novel	NA	Pathogenic
*NR5A1*	16	p.C73W	Het	Novel	NA	Pathogenic
*AMHR2*	17,18 (siblings)	p.R463H/p.R471H	Compound Het	Novel/Novel	NA/NA	Pathogenic
*HSD17B3*	19	p.E215D/c.277+4A>T	Compound Het	0.00009289 (European)/0.0006822 (European)	NA/Pathogenic (208587)	Pathogenic
*HSD17B3*	20	p.M47V/p.V243fs	Compound Het	0.00008516 (European)/Novel		Pathogenic
*MYRF*	21	p.N105D	Het	Novel	NA	Likely Pathogenic
*MYRF*	22	c.2572+1G>A	Het	Novel	NA	Likely Pathogenic
*ANOS1*	23	p.Gln586	Hemi	Novel	NA	Pathogenic
*ANOS1*	24, 25 (cousins)	p.Gln57fs*	Hemi	Novel	NA	Pathogenic
*FGFR1*	26	c.179_208del; p.Asp60_Asp69del	Het	Novel	NA	Likely Pathogenic
*WT1*	27	p.S255L	Het	Novel	NA	Likely Pathogenic
*DHX37*	28	p.R674Q	Het	Novel	NA	Pathogenic
*SRD5A2*	29	p.Y91H/p.G13R	Compound Het	0.0001913 (other)/Novel	Pathogenic (492899)/NA	Pathogenic
*GATA4*	30	p.G12R	Het	0.00003898 (European)	Uncertain significance (644697)	Likely pathogenic
*TBCE*	31	c.100+1G>A	Homozygous	Novel	NA	Likely pathogenic
*CYP19A1*	32	p.R457X/p.Leu353fs	Compound Het	0.00009800 (South Asian)/0.000008829 (European)	NA/Pathogenic (653853)	Pathogenic
*CACNA1A*	33	p.I219V	Het	Novel	NA	Pathogenic
*GLI2*	34	p.E1577K	Het	0.0009526 (East Asian)	Likely benign; Uncertain significance (798233)	Likely pathogenic
**VUS, Pathogenicity by ACMG**						
**Gene**	**Patient**	**Variant**	**Zygosity**	**MAF (population)**	**ClinVar (Variation ID)**	**Preliminary clinical diagnosis**
*ABCD1*	15	p.M539V	Het	Novel	NA	46XY, U
*ESR2*	33	p.Y49C	Het	Novel	NA	46XY, ovotesticular DSD
*WT1*	35	p.T474T	Het	Novel	NA	46XX, testicular DSD
*CBX2*	36	p.R135Q	Het	Novel	NA	46XY, GD
*POMC*	37	p.A100delinsSSGSSGA	Het	0.0003894 (African)	NA	46XY, TRS
*HSD17B13*		p.S165X/p.S201X	Compound het	Novel/Novel	NA/NA	
*SPRY4*	38	p.L283M	Het	Novel	NA	46XY, GD
*FLRT3*		p.T61R	Het	Novel	NA	
*FANCL*		p.Thr372fs	Het	0.006795 (Ashkenazi/Jewish)	Benign; Pathogenic; Uncertain significance (210988)	
*DHX37*	39	p.G1030E	Het	Novel	NA	46XY, TRS
*SRCAP*	40	p.S1747delinsSLAPAPP	Het	Novel	NA	46XY, U, AG
*WDR34*	41	p.Q158X	Het	0.00004054 (European)	Pathogenic (97044)	46XY, U, AG
*WDR35*		p.T1020R	Het	0.001015 (European)	Uncertain significance​ (333372)	
*DYNC2H1*		p.S2281C	Het	0.00009968 (Ashkenazi/Jewish)	NA	
*CDT1*		p.R138W	Het	0.0003477 (European)	Uncertain significance​ (1044530)	
*TRAF3IP1*		p.I386V	Het	Novel	NA	
*SOX4*		p.A275V	Het	Novel	NA	
*CCDC141*	42	p.Y1081_G1082delinsX	Homozygous	Novel	NA	46XY, U, AG
*WDR11*	43	p.F1150L/p.V356I	Compound het	0.008582 (Ashkenazi/Jewish) 0.002965 (European)	Likely benign; Uncertain significance (68842) Likely benign (695236)	46XY, U, AG
*MYRF*		p.A315V	Het	0.0001634 (South Asian)	NA	
*ANOS1*		c.856+3C>T	Hemi	Novel	NA	
*CCDC141*		p.D767N	Het	0.006612 (European Finnish)	Likely benign (638188)	
*FSHR*	44	p.S498R/p.S524R	Compound het	Novel/Novel	NA	46XY, TRS
*AMH*	45	p.D288E	Het	0.002311 (European)	NA	46XY, PAIS
*HESX1*	46	p.V129I	Het	0.001744 (Ashkenazi/Jewish)	Likely benign; Uncertain significance (26773)	46XY, U
*POU1F1*		p.D10G	Het	Novel	NA	
*LHCGR*	47	p.540_540delF	Het	0.00002892 (Latino)	NA	46XY, U, AG
*CYP19A1*	48	c.451+2T>A	Het	Novel	NA	46XY, U, AG
*POU1F1*	49	p.D10G	Het	Novel	NA	46XY, U, AG
*GNRHR*		p.P146S	Het	0.004587 (other)	Likely benign; Uncertain significance (449413)	
*GATA4*	50	p.P163S	Het	0.0006612 (East Asian)	Pathogenic; Uncertain significance (30099)	46XX, U, AG
*GALT*	51	p.P66L	Het	0.00006972 (European)	Uncertain significance​ (25136)	46XX, PH
*TACR3*		p.A449S	Het	0.0003724 (European)	Uncertain significance​ (436936)	
*LETM1*		c.1200+4C>T	Het	0.0002957 (European)	NA	
*NIN*		p.K2057Q	Het	Novel	NA	
*STAR*	52	p.D163N	Het	0.00008689 (Latino)	NA	46XY, GD (seminoma)
*SAMD9*	53	p.R1040C	Het	0.00009308 (European)	NA	46XY, TRS, Severe immunodeficiency
*STAT1*		c.1632+6G>A	Het	0.0003951 (European)	Benign; Uncertain significance (333271)	
*IKBKB*		p.A755S	Het	0.001372 (South Asian)	Likely benign (746609)	
*TAP2*		p.L59delinsLKLRGLL	Het	Novel	NA	
*HSD3B2*	54	p.A167V	Het	0.003724 (South Asian)	Benign; Uncertain significance (724290)	46XY, GD (gonadoblastoma)
*FANCM*		p.Ser497fs	Het	0.000008804 (European)	NA	
*PTCH1*	55	p.T627M	Het	Novel	NA	46XY, U, AG
*LZTR1*		p.M695I	Het	Novel	NA	
*HSD17B3*	56	p.G97A	Het	0.00001758 (European)	NA	46XY, U, AG
*LHCGR*	57	p.Y113N	Het	0.006076 (Ashkenazi/Jewish)	Benign; Likely benign; Uncertain significance (336469)	46XY, CAIS
*LHX3*		p.A3V	Het	0.001396 (European)	Likely benign; Uncertain significance (279836)	
*GNAS*		p.A426P	Het	0.00008206 (European)	Likely benign (931358)	
*PAX4*		p.C282R	Het	0.002110 (European)	Likely benign (436159)	
*HHAT*	58	p.V399fs	Het	Novel	NA	46XY, GD (dysgerminoma)
*AMH*	59	p.T22A	Het	0.00006401 (Latino/Admixed American)	NA	46XY, TRS
*CHD7*	60	p.M2527L	Het	0.003603 (European)	Likely Benign (158317)	46XY, TRS
*ANOS1*		c.256-2A>T	Hemi	Novel	NA	
*WDR11*	61	p.A435T	Het	0.0004573 (South Asian)	Pathogenic (37309)	46XY, GD (gonadoblastoma)
*WDR11*	62	p.A435T	Het	0.0004573 (South Asian)	Pathogenic (37309)	46XY, TRS
*CHD7*		p.M340V	Het	0.009463 (Ashkenazi/Jewish)	Likely Benign (95773)	
*POR*		p.V348I	Het	0.00004061 (European)	Uncertain significance (910412)	

NA, Not Applicable.

Eleven patients from 10 families carried hemizygous pathogenic variants in the *AR* gene. Of the nine *AR* variants associated with XY DSD, eight have not been reported to cause either PAIS or CAIS and they are absent from public SNP databases (**Table 2**). Eight of the variants are missense and one is an essential splice donor site. Of the eleven patients, five were classified as CAIS and seven registered as female. Two girls were defined as PAIS due to clitoromegaly. Six females had a gonadectomy between 6 and 16 years old, and of these, one girl was diagnosed with a sertolioma at 14 years (**Table 1**). Four of the eleven patients were registered as male with a PAIS phenotype. Cases 7 and 8 are siblings (*AR* p.I843T variant), where one was raised as a boy and the other as a girl. The former presented with pubertal delay and micropenia. One patient with *AR* p.Leu839Ile had atypical genitalia at birth and was registered as female, but child’s gender was reassigned to male at 11 months.

The second most common genetic cause of DSD in the XY cohort are variants in the *NR5A1* gene (**Tables 1**, **2** and **Figure 1B**). Five individuals from 4 families carried heterozygous pathogenic variants in the *NR5A1* gene. Two were raised as boys and three as girls. All variants are novel and consists of three missense variants, all located within the zinc-finger DNA-binding domain (G35D, p.C73W, p.C73Y; **Table 1**). The other splice site variant (c.244+1G>T) was carried by twin boys born after IVF treatment. At birth they presented with bilateral cryptorchidism, perineal hypospadias and micropenia. Family history indicated that the father and grandfather had a similar DSD phenotype at birth and underwent surgery of the external genitalia and cryptorhidism correction. The *NR5A1* variant p.G35D was carried by a girl who presented with clitoromegaly and urogenital sinus at birth. Before karyotyping she was diagnosed with CAH and glucocorticoids were prescribed, but were stopped at the age of 4 years. A follow up examination after receiving of the WES result at 9 years revealed signs of moderate primary hypocorticism (ACTH 122 pg/ml (normal range 6-55), cortisol daily urine 42.4 μg/day (normal range 58-403). Two girls who carried the *NR5A1* p.C73Y and p.C73W variants presented at birth with clitoromegaly and the gonads were located in the inguinal canals.

Two siblings (cases 17 and 18) with compound heterozygous variant in the *AMHR2* gene (p.R463H/p.R471H) were reported previously (15).

Furthermore, two girls (cases 19, 20) presented with compound heterozygous variants in the *HSD17B3* gene. One presented at 7 months due to the presence of bilateral inguinal hernias and surgery was performed. At 10 years old US of the pelvic organs and inguinal canals revealed the absence of the uterus and the location of the gonads in the inguinal canals. She carried rare *HSD17B3* variants (p.E215D/c.277+4A>T). A second girl with rare and novel *HSD17B3* variants (p.M47V/p.V243fs) presented at birth had ambiguous genitalia (scroto-labial folds, perineal hypospadias, urogenital sinus) and the location of the gonads in the scroto-labial folds.

Two individuals carried LP and novel *MYRF* variants. A boy (case 21, p.N105D) presented at birth with ambiguous genitalia and was registered as a female. The child’s gender was reassigned to male after the discovery of a 46,XY karyotype at 1 month. There were no other somatic phenotypic anomalies. A girl (case 22) carries a heterozygous *MYRF* loss-of-function (LOF) variant (c.2572+1G>A). She presented at 14 years of age with primary amenorrhea, lack of secondary sexual characteristics and high-grade hypermetropia. She also had PH, hirsutism and vaginal hypoplasia. US of the pelvic organs indicated a hypoplastic uterus and right migratory gonad (from the abdominal cavity to the inguinal canal). The left gonad was absent. She underwent a gonadectomy and vaginoplasty with clitorectomy at the age of 26 and 29 years old respectively.

Four boys were diagnosed with hypo-/anosmic hypogonadotropic hypogonadism (HH). In three of the boys two novel hemizygous P variants were identified in the *ANOS1* gene (**Table 2**). Case 23, carrying an *ANOS1* c.1756C>T (p.Gln586) variant, presented with hypogenitalism (micro-orchidism, testis volume 0.65 ml) and hyposmia at 17 years old. Serum inhibin B and AMH levels were normal. Standard short HCG test (three injections for three consecutive days of 1500/m^2^ IU of HCG) was negative, however long HCG test was positive (doubled total testosterone level on day 11^th^), but this did not result either in further testosterone increasing nor testis enlargement based on the US data and clinical examination 19 days after the test. Considering the absence of a history of cryptorchidism and the normal inhibin B and AMH levels, the child underwent a subsequent treatment with Follitropin alfa, which led to the enlargement of the testicular volume by 77% and the penile, but testosterone levels did not increase. The MRI of the brain confirmed a hypoplasia of the olfactory bulbs. Two cousins with hyposmia (cases 24-25) carried an *ANOS1* c.171_181del (p.Gln57fs*) variant inherited from their mothers, and bilateral inguinal cryptorchidism developed after 6 years of age in a proband, but in his cousin right-sided inguinal cryptorchidism and micropenia presented at 1 year of age and he had orchidopexy at 5 and 9 years without an effect. Both siblings did not receive treatment until 14 and 15 years respectively. The GnRH stimulation test confirmed HH and a standard short HCG test was positive in both patients. Serum inhibin B and AMH levels were normal. Treatment with HCG at 14 and 15 years of age lead to the descent of the testes into the scrotum and increased testosterone levels after 10 weeks of treatment in both patients. Case 26 with an *FGFR1* (c.179_208del; p.Asp60_Asp69del) pathogenic variant presented at 2 years with micropenia that was first observed at birth. A persistent bilateral inguinal cryptorchidism appeared at 4 years. Serum inhibin B and AMH levels were low. The GnRH stimulation test confirmed HH and a standard short HCG test was negative. The child also has pulmonary hypoplasia and bronchiectasis. Subsequent treatment with HCG was ineffective and he underwent an orchidopexy at 9 years. His mother and grandmother reported the presence of anosmia. At the age of 9 years new clinical features (primary hypoparathyroidism, hypothyroidism and epilepsy) were noted.

P/LP variants in the genes *WT1, DHX37, SRD5A1, GATA4, TBCE, CACNA1A* and *GLI2* were identified in single individuals (**Tables 1**, **2**). A 46,XY girl with normal renal function carried a heterozygous *WT1* p.S255L variant that was inherited from her healthy mother. She presented at 12 years with primary amenorrhea and the absence of secondary sexual characteristics. US of the inguinal canals revealed the presence of gonads and she underwent gonadectomy. The *DHX37* variant was reported previously (5). Case 29, who carries compound heterozygous variants in the *SRD5A2* gene, presented at 14 years with primary amenorrhea, clitoromegaly and absence of secondary sexual characteristics. US of the inguinal canals revealed the presence of gonads with subsequent gonadectomy. Within the cohort a single individual carried a LP, rare missense variant in the *GATA4* gene (p.G12R) that was inherited from her healthy mother. The girl presented at the age of 3 years with significant growth retardation (> - 2SD) and her karyotype revealed a minimal mosaicism (93.3% of nuclei with locus Yq12) (46, XY. nuc ish Xp11.1- q.11.1) (DXZ1 *1), Yq12 (DYZ1-) [25]/Xp11.1-q.11.1 (DXZ1*1), Yq12 (DYZ1 * 1) [375]). Further examination revealed PH, and US confirmed the presence of the uterus and gonads in the abdominal cavity. Gonadectomy was done at the age of 4 years due to a significant growth retardation (> - 2SD) and the need to initiate treatment with growth hormone. Ultrasound of the heart (EchoCG) was normal in both the mother and child carrying the *GATA4* variant. Case 31, a 46,XY boy, was born with SGA and inguinal bilateral cryptorchidism. He carries a homozygous splice site variant (c.100+1G>A) in the *TBCE* gene. Biallelic variants of *TBCE* are associated with Hypoparathyroidism-Retardation-Dysmorphism Syndrome and Kenny-Caffey Syndrome (OMIM 604934). He underwent two stages of surgery (at 3 and 4 years), after which however, the right testis remained in the inguinal canal and the left testis was removed because of its atrophy. He also had cognitive impairment, dysmorphic features and recurrent infections. At the age of 14 years, further examination showed a PH and dwarfism (height -2.8 SD) with the normal clonidine test, but low IGF-1 level (>- 2SD). Final gonadectomy was done at the age of 15 years.

Two of the patients in the cohort carried variants that contributed to their phenotype, but the association of these variants with DSD is unclear. Case 33, is a 46,XY boy who presented at birth with bilateral cryptorchidism, scrotum hypospadias, micropenia and a double left kidney. He underwent gonadectomy in 2 stages because of ovotesticular DSD at 6 and 7 years. Histopathology indicated a germinogenic tumor and the child had subsequent chemotherapy. At 13 years the boy developed epilepsy. He carries a heterozygous *CACNA1A* p.I219V variant. Variants in the *CACNA1A* gene are associated with epilepsy (16), but to our knowledge variants in this gene are not associated with DSD. The second individual, case 34, is a female who presented with short stature, PH (FSH 111.1 mIU/ml, LH 24.2 mIU/ml), absence of the uterus and gonads and vaginal hypoplasia at the age of 12 years. The child also presented with congenital coarctation of the aorta, cleft palate, and facial dysmorphia. She carries a *GLI2* p.E1577K variant. Variants in this gene are associated with autosomal dominant holoprosencephaly 9 and Cooler-Jones syndrome. These syndromes are characterized by hypopituitarism with dwarfism, developmental delay, polydactyly and facial dysmorphia, and this can explain most of the phenotype of the girl (17). However, the DSD associated with *GLI2* variants are considered secondary to the pituitary anomalies and they are not associated with PH. Gonadectomy was done at the age of 13 years. After the gonadectomy and initiation of the replacement therapy with growth hormone therapy, she was subsequently found to have an additional structure near the uterus with a fluid component from a pelvic ultrasound, performed as part of this study. Subsequent surgery confirmed the presence of a detached rudimentary uterine horn.

### 46,ХХ DSD Cohort

**Figure 2:**
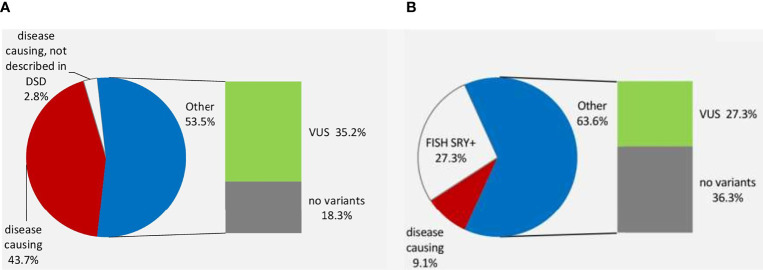
The genetic diagnosis of 46, XY DSD **(A)** and 46,XX DSD **(B)**.

A lower genetic diagnostic yield of WES was found for 46,XX DSD compared with 46,XY DSD cohort (12.5% and 46.5% accordingly), (**Figures 2A, B**). In the former group 61.9% (n=13) were raised as girls and 38.1% (n=8) were raised as boys. Three of the eight boys carried the *SRY* gene, which explains the phenotype. Two of the three 46,XX *SRY*+ boys had cryptorchidism and two had severe somatic anomalies (severe myopathy in one and cognitive deficiency and anorectal atresia in another). Of the patients with 46,XX DSD with female presentation (n=13), six patients had a PH without virilization, whilst others had signs of androgen excess (Prader 3-5). WES performed for one boy and seven girls (n=8) and identified rare biallelic and pathogenic *CYP19A1* variant (p.R457X/p.L353fs) in one child (case 32) who presented with ambiguous genitalia and was registered as a male. At the age of 8 months, following the discovery of a 46,XX *SRY*- negative karyotype the child’s gender was reassigned to female. At one year of age, she was diagnosed with fluorocolpos, with a spontaneous regression. Repeated hormonal investigations at the age of 5 years old indicated PH (FSH 22 mIU/ml). At 10 years the child underwent plastic surgery for the urogenital sinus. At this time the girl had elevated FSH level (34.4 mIU/ml).

VUS variants were found in 28/79 patients (35.4%) and their contribution to DSD is currently unknown, however two patients with P variants (case 15 and 33) also carry VUS and potentially can have a digenic disease (**Table 2** and **Figures 2A, B**).

## Discussion

A single study reported a diagnostic yield of 35% following WES on a large DSD cohort (18). Other studies, on smaller patient numbers using either tNGS or WES approaches, have reported a diagnostic yield of 23%-69.5% (11, 13, 19–26). The difference in yield is attributed to the makeup of patients’ cohorts included in each study. The highest diagnostic rate (64%) was found by Laino et all (11), presumably due to the inclusion of patients with CAH. In all studies a higher diagnostic yield was found for 46,XY DSD patients compared to 46,XX (13, 20, 27, 28). In our study we found that the diagnostic yield after WES was higher than previously reported (18) for either P/LP variants and VUS (43% vs 35% and 35.4% vs 15% accordingly). Pathogenic variants in the *AR* gene (n=11) and the *NR5A1* gene (n=5) were the most common cause (47%) of DSD among patients with a genetic diagnosis (**Table 1** and **Figure 1B**). 83.3% of all P/LP variants were novel.

Two cases with variants in *DHX37* contributed to the discovery of this gene as a new cause of DSD (5), making this approach especially promising for DSD patients that do not have a genetic etiology. The majority of patients in the cohort are unexplained, but 35.4% carry one or more VUS that require careful ongoing evaluation in order to establish causality. For example, a 46,XX DSD male with a *de novo* synonymous variant in *WT1* p.T474T (case 34), is considered a good candidate for DSD based on previous reports (29), but requires further studies to establish pathogenicity.

An important finding in this study are variants involving heterozygous *WDR11* and DSD. Heterozygous variants in *WDR11* are proposed as a cause of autosomal dominant CHH (30, 31), including two variants p.A435T and p.F1150L (30). Here, we identified two patients carrying the heterozygous p.A435T variant (cases 60 and 61), who presented with severe PH (46,XY female with GD and a gonadoblastoma and a 46,XY male with TRS). Both variants were inherited from their healthy mothers. A recent study identified biallelic LOF *WDR11* variants in association with a complex familial phenotype of microcephaly and intellectual disability (32). There were no reproductive phenotype reported in all affected individuals as well as carriers (32). Together with our data, this suggests that heterozygous *WDR11* variants are unlikely to cause CHH and questions their contribution to any reproductive phenotype. However further studies are needed to formally establish the role of *WDR11* in human disorders.

For the DSD patients in whom WES identified P/LP variants (n=34), the most common variants among males (n=15) were *AR* variants (n=4), HH with *FGFR1* and *ANOS1* (n=4, including two affected cousins), an *NR5A1* variant in twin boys (n=2) and *AMHR2* variants in siblings (n=2). In patients who were registered as females (n=19), the most common etiology of DSD (36.8%) were *AR* gene variants (n=7). This differs from published data (7, 8), and may be due to the exclusion of patients with classical CAH from our study. According to the international Consensus (1), registration of female sex is recommended for 46,XX patients with CAH, or CAIS and 46,XY patients with LH receptor deficiency. Registration of male sex is recommended for 5α-reductase deficiency, since 60% of them later self-identify as males, and for 17β-HSD3 deficiency, because more than 50% of patients later self-identify as male. In our cohort all patients with 46,XY DSD and pathogenic *HSD17B3* and *SRD5A2* variants were raised as females. It is generally believed that in the process of the patient’ sex registration the potential quality of sexual life is a key factor, as available evidence suggests that genital anatomy is less influenced than other important factors associated with interpersonal relationships (33).

The WES diagnostic rate differed between the DSD subcategories. This was highest in patients with a clinical suspicion of Kallmann syndrome (n=100%) and with DASA (83.3%), which corresponds to previous findings (13). In contrast, the diagnostic yield was lower in GD patients (35.7%) as well as in the DSD group of undefined etiology with AG (U, AG) (22.2%), and was lowest in the TRS cohort (8.3%). VUS were the most frequent in the U, AG and TRS patients (35.7% and 25% respectively). No patient with 46,XX PH (n=5) carried with P/LP variants. The number of 46,XX DSD patients was lower than 46,XY in the cohort since CAH patients were excluded. The diagnostic yield was higher for 46,XY compared to 46,XX DSD groups (46.5% and 12.5% respectively), which is similar to that reported elsewhere (13, 20, 27, 28). Of the eight 46,XX DSD patients who underwent WES, only a single case with *CYP19A1* was confirmed.

A significant number (12/34) of patients with a genetic diagnosis had atypical clinical presentations. An example is the presence of hypospadias in both siblings with PMDS (cases 17-18), where the uterus was not solidly attached to the testis in one of patients (15). This raises the question of how many cases with a simple bilateral inguinal cryptorchidism due to *AMH/AMHR2* variants may be missed. *GATA4* and *WT1* variants were observed in the absence of neither heart or kidney anomalies respectively, inherited from their unaffected mothers. This may be due to incomplete penetrance, or oligogenic mechanisms (34). The *MYRF* LOF variant reported here in a child with gonadal dysgenesis and nanophthalmos is, to our knowledge, the first case with this combination of phenotypes. Other examples of atypical presentation include a heterozygous LOF *TBCE* variant in a syndromic form of 46,XY DSD. Pathogenic variants in *TBCE* are associated with neurodevelopmental syndromes, hypoparathyroidism-retardation-dysmorphism, Kenny-Caffey syndrome (35, 36) and with hypopituitarism (including GH insufficiency, hypocortisolemia and CHH) (37). However, probable PH was also described in association with extreme growth failure, dysmorphic features and hypoparathyroidism (38). In our case hypoparathyroidism was absent in the presence of PH (FSH 85.9 mIU/ml). Variants in the *CYP19A1* gene cause aromatase deficiency and are considered a rare recessive disease with about 40 cases of aromatase deficiency reported (39). We report biallelic variants in *CYP19A1* with an atypical presentation consisting of virilization in the child (development of clitoromegaly and the formation of the urogenital sinus), an absence of ovarian cysts, and FSH and LH levels that increased after 5 years of age. The individual harboring a *GLI2* variant had a bicornuate uterus with non-communicating rudimentary horn, which has not been previously described in 46,XY DSD patients to our knowledge. An atypical clinical presentation was found in two boys with HH. Pathogenic variants in *ANOS1* and *FGFR1* cause Kallmann’s syndrome (40). In our cohort, four patients (cases 24-25) had a classical clinical picture of Kallmann’s syndrome, but a boy (case 23) with the *ANOS1* variant p.Gln586 had increased serum testosterone levels but did not have enlargement of testis after the long HCG test with normal inhibin B and AMH levels. The prepubertal testicular volume (<4 ml), low serum inhibin B concentration and a history of cryptorchidism are described as negative predictors of stimulating treatment response (41) as we observed in case 26, but in case 23 both treatment modalities (Follitropin alfa and HCG) were ineffective where only micro-orchidism was present. This suggests that a small testicular volume by itself can be considered as unfavorable predictor, which differs from published data (42) and ‘primary’ hypogonadism in GnRH non-responders with *ANOS1* variants should not be excluded (43). However, two cousins with an *ANOS1* LOF variant and with a history of either untreated cryptorchidism, small testicular volume (<4 ml) but normal AMH and inhibin B levels had a good response to HCG treatment. A boy (case 26) with HH who carried an *FGFR1* variant had novel extragenital features, including pulmonary hypoplasia, bronchiectasis, primary hypothyroidism, hypoparathyroidism and epilepsy. Activating variants in *FGFR1* were reported in patients with osteoglophonic dysplasia and hypophosphatemia, moreover in one family all family members with *FGFR1 p.Y372C* died due to affected respiratory function (44). Further studies of the possible impact of *FGFR1* on pulmonary and other extragenital diseases in patients with HH are needed. Also, two patients with *CACNA1* and *GLI2* variants presented with PH that has not been previously reported. Overall, 10/32 of patients with P/LP variants in genes known to cause DSD, had atypical clinical presentations.

Almost all DSD patients in our cohort had non-specific clinical signs (e.g., ambiguous genitalia, clitoromegaly, urogenital sinus, hypospadias, inguinal hernias, cryptorchidism, etc.), with non-specific changes in hormonal levels. WES is recommended for these patients, since predicting which gene may be involved is challenging. Determining the etiology is important to assess the risk of gonadal malignancy, since these patients are at an increased risk (1, 33). In our cohort nine 46,XY DSD individuals had a malignant gonadal tumor. Of these only two patients carried P variants (*AR* and *CACNA1A*; 22.2%). Overall, of the nine 46,XY patients, eight were female (with unambiguously female phenotype, i.e. with severe undervirilization) with the exception of three patients who presented with clitoris enlargement at different age (at the earliest at 4 years) and one with *AR* gene variant with location of gonads in the inguinal canals. All tumors were seminomatous and chemotherapy was required for 3/9 patients. In 8/9 patients the gonads were located in the abdominal cavity (7 females/1 male). Although the risk of malignancy is considered low in ovotestis and CAIS and before the pubertal age (1, 33), two of these patients had malignant tumors at an early age. In the entire cohort, three patients had gonadal malignant tumors before 10 years of age. This data supports previous studies where a combination of severe undervirilization and location of the gonads in the abdominal cavity are the risk factors for gonadal tumors (45, 46). However, their early age of onset in 33.3% of patients in our study is also rare finding.

## Conclusions

The most common genetic causes of DSD in this study are P/LP variants in the *AR* and *NR5A1* genes (20.2% of entire WES cohort and 47% among patients with a genetic diagnosis). Remarkably, almost 84% of all P/LP variants have not been reported elsewhere and a significant number of patients (35.3%) carrying these variants had atypical clinical presentations. This indicates that WES is the approach of choice to obtain a genetic diagnosis in these conditions, which can be difficult to define based on the clinical presentation and hormonal data. Our data also question the contribution of *WDR11* variants to CHH.

## Data Availability Statement

The data presented in the study are deposited in the ClinVar (https://www.ncbi.nlm.nih.gov/clinvar/) repository, accession numbers (VCV000492789, VCV000974911, VCV000658124, VCV001202584, VCV001202585, VCV001202591.1, VCV001205840.1, VCV001202603.1, VCV000935575, VCV001202587, VCV001202586, VCV001202588, VCV001202589, VCV000929443, VCV000981464, VCV000631595.5, VCV001199399.1 and VCV001199405.1).

## Ethics Statement

The studies involving human participants were reviewed and approved by Local ethical committee of Ukrainian Scientific and Practical Center of Endocrine Surgery, Transplantation of Endocrine Organs and Tissues of the Ministry of Health of Ukraine, (№ 34, 26.12.2016). Written informed consent to participate in this study was provided by the participants’ legal guardian/next of kin.

## Author Contributions

EG performed a clinical investigation of patients at the initial stage and follow-up, was responsible for conception and design of the study, data acquisition, preparation of the manuscript, finding relevant references, and final approval of the manuscript. NZ performed a clinical investigation of patients at the initial stage and follow-up; designed the analyses; reviewed and edited the manuscript. YS performed a clinical investigation of 15 patients at the initial stage. JB-T, AB, and KM performed and interpreted genetic testing; conceptualized and designed the study; and critically reviewed and revised the manuscript. KM is the guarantor and approved the final manuscript as submitted.

## Funding

This work is funded in part by a research grant from the European Society of Pediatric Endocrinology, and by the Agence Nationale de la Recherche (ANR), ANR-10-LABX-73 REVIVE, ANR-17-CE14-0038-01, ANR-19-CE14-0022 and ANR-19-CE14-0012 and by the Ministry of Health of Ukraine (0117U003036).

## Conflict of Interest

The authors declare that the research was conducted in the absence of any commercial or financial relationships that could be construed as a potential conflict of interest.

## Publisher’s Note

All claims expressed in this article are solely those of the authors and do not necessarily represent those of their affiliated organizations, or those of the publisher, the editors and the reviewers. Any product that may be evaluated in this article, or claim that may be made by its manufacturer, is not guaranteed or endorsed by the publisher.
